# Supplementary material for: The interactome of intact mitochondria by cross-linking mass spectrometry provides evidence for coexisting respiratory supercomplexes
Source: Mol Cell Proteomics. 2017 Dec 8;17(2):216–32. doi: 10.1074/mcp.RA117.000470 (PMC5795388; doi:10.1074/mcp.RA117.000470)
Supplement: Supplemental Data [file supp_RA117.000470_133922_0_supp_23978_fzffwf.zip › spectra_annotation/mito_spectra_annotation/315-1-1-1-7-1.pdf]

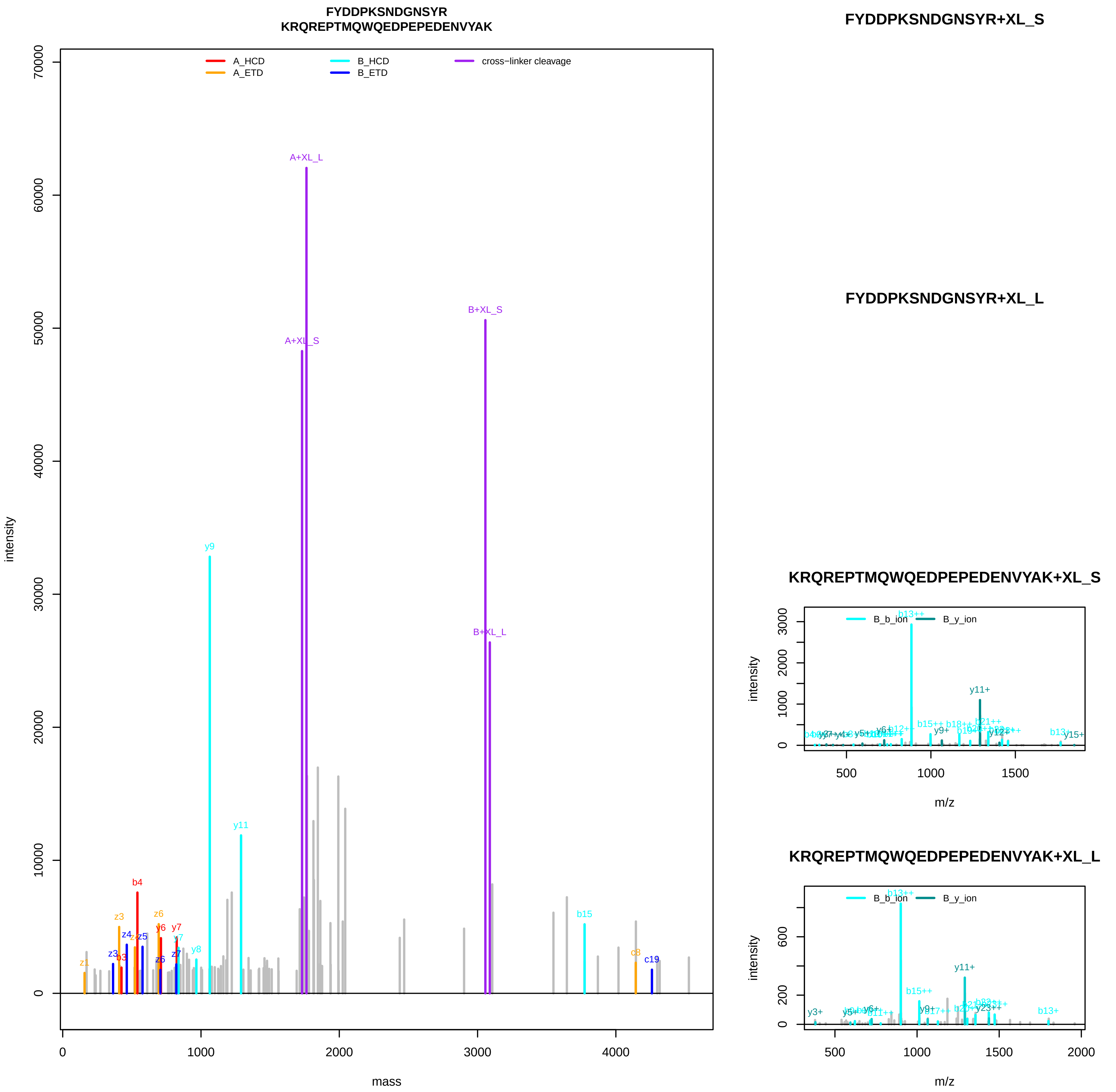
FYDDPKSNDGNSYR
KRQREPTMQWQEDPEPEDENVYAK
A_HCD
A_ETD
B_HCD
B_ETD
cross−linker cleavage
intensity
mass
A+XL_L
A+XL_S
B+XL_S
B+XL_L
y9
y11
b4
b15
c8
c19
z1
z3
z4
z5
z6
z7
b3
y6
y7
y8
0
10000
20000
30000
40000
50000
60000
70000
1000
2000
3000
4000
FYDDPKSNDGNSYR+XL_S
FYDDPKSNDGNSYR+XL_L
KRQREPTMQWQEDPEPEDENVYAK+XL_S
B_b_ion
B_y_ion
b13++
y11+
y9+
y12+
b15++
b18++
b21++
b13+
y15+
y5+
y6+
b12++
m/z
500
1000
1500
KRQREPTMQWQEDPEPEDENVYAK+XL_L
y3+
y23++
b11++
b17++
2000
200
600
